# FOXO3a Potentiates hTERT Gene Expression by Activating c-MYC and Extends the Replicative Life-Span of Human Fibroblast

**DOI:** 10.1371/journal.pone.0101864

**Published:** 2014-07-07

**Authors:** Shuntaro Yamashita, Kaori Ogawa, Takahiro Ikei, Tsukasa Fujiki, Yoshinori Katakura

**Affiliations:** 1 Graduate School of Systems Life Sciences, Kyushu University, Higashi-ku, Fukuoka, Japan; 2 Graduate School of Bioresources and Bioenvironmental Sciences, Kyushu University, Higashi-ku, Fukuoka, Japan; 3 Faculty of Agriculture, Kyushu University, Higashi-ku, Fukuoka, Japan; INSERM UMR S_910, France

## Abstract

In our previous studies, we reported that SIRT1 prevents cellular senescence in human fibroblast, and that SIRT1-induced inhibition of cellular senescence is due to enhanced hTERT gene expression. In this study, we investigate the molecular mechanisms behind SIRT1-induced potentiation of hTERT transcription and show that FOXO3a functions downstream of SIRT1 and prevents the induction of cellular senescence by enhancing hTERT gene expression. Furthermore, we found that FOXO3a-induced potentiation of hTERT gene expression is regulated in a c-MYC/E-box dependent manner. In addition, we found that FOXO3a binds to the novel binding element in the c-MYC promoter, and this interaction activates the transcription of the c-MYC gene. The resulting increase in c-MYC leads to higher levels of c-MYC recruited to the hTERT promoter and, in turn, activates hTERT gene expression. Taken together, this pathway might constitute the molecular basis for the anti-senescence effects of SIRT1 and FOXO3a.

## Introduction

SIRT1 is a multifunctional NAD^+^ dependent protein deacetylase that is involved in a wide variety of cellular process. Previous reports have shown that the activation of SIRT1 is beneficial in several age-related diseases, particularly those associated with metabolic dysregulation, through the activation of tissue/cell specific transcription factors [1]–[3]. Among these functions, several labs, including our own, have reported that SIRT1 overexpression antagonizes cellular senescence and that SIRT1 inhibitors induce cellular senescence in human cells, implicating a role of SIRT1 in the inhibition of cellular senescence [4]–[6]. In our previous study, we investigated the molecular mechanisms of SIRT1-induced inhibition of cellular senescence and demonstrated that SIRT1-induced inhibition of cellular senescence is elicited by potentiating the transcription of the human telomerase reverse transcriptase (hTERT) gene, which encodes the enzyme responsible for maintaining the integrity of chromosomal ends.

hTERT is known to play a crucial role in cellular immortalization, tumorigenesis, and the progression of cancer. Transcriptional regulation of the hTERT gene is a major mechanism underlying the cancer-specific activation of telomerase, and a large number of transcription factors have been identified to directly or indirectly regulate the hTERT promoter [7], [8]. In addition, we previously reported that cellular senescence-inducing factors, such as TAK1, PKC-δ, and cellular senescence-inhibiting factor SIRT1, regulate hTERT transcription. This suggests that understanding the mechanisms behind the transcriptional regulation of hTERT is required to elucidate the molecular mechanisms of cellular senescence [6], [9], [10].

In our previous study, we demonstrate that the cellular senescence-inhibiting factor SIRT1 potentiates the transcription of the hTERT gene. Here, we refine the molecular mechanisms for SIRT1-induced enhancement of hTERT transcription.

## Materials and Methods

### Cell lines

Normal human umbilical cord fibroblasts (HUC-F2) were obtained from Riken Bioresource Center (Tsukuba, Japan). Cells were maintained in Dulbecco’s Modified Eagle’s Medium (DMEM, Nissui, Tokyo, Japan) supplemented with 10% fetal bovine serum (FBS, Invitrogen, Carlsbad, CA).

### Retrovirus production and transduction

Viral supernatants were produced after transfection of 293T cells with pGag-pol, pVSV-G, and individual expression vectors (pBABE-puro-FOXO3aWT, pBABE-puro-FOXO3aTM, pBABE-puro-SIRT1, or mock) using the HilyMax reagent (Dojindo, Kumamoto, Japan) as previously described [6]. The cells were cultured at 37°C in DMEM supplemented with 10% FBS for 24 h. The medium was replaced with DMEM supplemented with 2% FBS and incubated for an additional 24 h. Viral supernatant was collected and supplemented with 10% FBS and 10 µg/mL polybrene (Merk Millipore, Billerica, MA). The target cells were infected with this viral supernatant for 24 h at 37°C. After infection, the cells were selected with 3 µg/mL puromycin (Enzo Life Sciences, Farmingdale, NY) for 3 days. Expression level of retrovirus transgene was shown in Figure S1 and S3.

### Short hairpin RNA (shRNA)

The oligonucleotides that contain the siRNA-expressing sequence targeting FOXO3a were annealed (shFOXO3a-1 top: 5′-GATCCCCGTGGAGCTGGACCCGGAGTTTCGAAGAGACTCCGGGTCCAGCTCCACTTTTTA-3′, shFOXO3a-1 bottom: 5′-AGCTTAAAAAGTGGAGCTGGACCCGGAGTCTCTTCGAAACTCCGGGTCCAGCTCCACGGG-3′; shFOXO3a-2 top: 5′-GATCCCCGAGCTCTTGGTGGATCATCTTCGAAGAGGATGATCCACCAAGAGCTCTTTTTA-3′, shFOXO3a-2 bottom: 5′-AGCTTAAAAAGAGCTCTTGGTGGATCATCCTCTTCGAAGATGATCCACCAAGAGCTCGGG-3′), and cloned into the pSUPER.retro vector (OligoEngine, Seattle, WA). Viral supernatant was prepared as described above, and the cells were infected with this viral supernatant for 24 h at 37°C. Transduced cells were selected with 3 µg/mL puromycin for 3 days. Efficiency data of knockdown experiment was shown in Figure S2.

### Senescence-associated β-galactosidase (SA-β-Gal) assay

The SA-β-Gal assay was performed according to the method described by Dimiri et al. [11]. Staining was performed at 37°C for 12 h.

### Promoter assay

Reporter containing the DAF-16 family protein-binding element (DBE) was generated by ligation of annealed oligonucleotide ((TTGTTTAC)_6_) to the pGL3-Promoter (Promega, Madison, WI) and named pGL3-DBE [12]. pGL3-Basic (Promega) containing the hTERT core promoter (−289 to −25) (phTERTp-289) and mutant hTERT core promoter with two disrupted E-box elements (phTERT-289EM) were used as reporter vectors [9]. A reporter vector containing the human c-MYC promoter (pDel-1-Luc) was obtained from Dr. Bert Vogelstein [13]. Two putative FOXO binding elements in the c-MYC promoter region (−1797 to −1790 and −330 to −323) were mutated from TTGTTTTC to TCCCCTTC and CTGTTTAC to CCCCCTAC by site-directed mutagenesis [14]. HUC-F2 (2.5×10^5^ cells) cells were seeded onto a 24-well dish and, the next day, were transfected with the reporter and effector constructs using the HilyMax reagent according to the manufacturer’s protocol. After 48 h, a luciferase assay was performed using the Dual-Luciferase Reporter Assay System (Promega). To evaluate the transcriptional activation ability of c-MYC, we generated GAL4-c-MYC, which contains a full-length human c-MYC cDNA (amino acids 1–440) fused to the DNA-binding domain of the yeast transcription factor GAL4 (pFA-CMV, Agilent Technologies, Santa Clara, CA) [6]. Luciferase assays were performed using GAL4-c-MYC and pFR-Luc (Agilent Technologies), a luciferase reporter vector containing five GAL4 binding sites.

### Adenovirus production and transduction

We produced recombinant adenovirus using the Adeno-X Expression System (Takara, Shiga, Japan) according to the manufacturer’s protocol. Recombinant adenoviruses were prepared by disrupting HEK293 cells (JCRB9068; HSRRB, Osaka, Japan) transfected with recombinant adenoviral DNA including SIRT1 or SIRT1-HY cDNA (Addgene, Cambridge, MA). All viruses were titrated using a method that measures the 50% tissue culture infectious dose (TCID_50_). HUC-F2 cells underwent adenoviral transduction after a 1-h infection at a multiplicity of infection (MOI) of 50.

### Quantitative RT-PCR (qRT-PCR)

RNA was isolated using the High Pure RNA Isolation Kit (Roche, Mannheim, Germany). cDNA was generated from the isolated RNA using the ReverTra Ace kit (Toyobo, Osaka, Japan). qRT-PCR was performed using the KAPA SYBR Fast qPCR Kit (KAPA Biosystems, Woburn, MA) and the Thermal Cycler Dice Real Time System TP-800 instrument, as described previously [6]. The samples were analyzed in triplicate, and the hTERT, and c-MYC levels were normalized to the corresponding β-actin level. The following PCR primers were employed: hTERT forward primer, 5′-CGTACAGGTTTCAC GCATGTG-3′ and reverse primer, 5′-ATGACGCGCAGGAAAAATG-3′; human c-MYC forward primer, 5′-CGGATTCTCTGCTCTCCTCGAC-3′ and reverse primer, 5′-CCTCCAGCAGAAGGTGATCCA-3′; human β-actin forward primer, 5′-TGGCACCCAGCACAATGAA-3′ and reverse primer, 5′-CTAAGTCATAGTCCGCCTAGAAGCA-3′.

### Telomere length measurement

Genomic DNA was extracted using QIAamp DNA blood Mini kit (Qiagen K.K., Tokyo, Japan). Telomere length was determined using qPCR as described previsouly [15]. DNA (35 ng) was used for each PCR reaction and PCR was carried out as described above. The primer sequences for human telomere (tel1 and tel2) and 36B4 were: tel1: 5′-GGTTTTTGAGGGTGAGGGTGAGGGTGAGGGTGAGGGT-3′; tel2: 5′-TCCCGACTATCCCTATCCCTATCCCTATCCCTATCCCTA-3′; 36B4u: 5′-CAGCAAGTGGGAAGGTGTAATCC-3′; 36B4d: 5′-CCCATTCTATCATCAACGGGTACAA-3′. The telomere signal was normalized to the signal from the single-copy gene to generate a relative telomere to a single copy gene ratio indicative of relative telomere length.

### Chromatin immunoprecipitation (ChIP) assay

A ChIP assay was performed using the ChIP assay kit (Merk Millipore, Billerica, MA) as described previously [9]. Briefly, cells were crosslinked with formaldehyde, and the cell pellet was resuspended in SDS lysis buffer. The sonicated chromatin solution was incubated overnight at 4°C with either the anti-c-MYC antibody (N-262; Santa Cruz Biotech., Santa Cruz, CA), anti-acetyl-histone H4 antibody (06–866; Merk Millipore), or FOXO3a antibody (#9467, Cell Signaling, Danvers, MA). Rabbit IgG (#3900S, Cell Signaling) was used as control. The immune complexes were collected using salmon sperm DNA/protein A agarose and eluted with elution buffer (1% SDS and 0.1 M NaHCO_3_), and the crosslinkage was reversed by heating at 65°C for 6 h, followed by treatment with proteinase K at 45°C for 60 min. The DNA was recovered by ethanol precipitation and used as a template for PCR to amplify the target site in the hTERT promoter and c-MYC promoter as previously described [9]. The utilized target sites and the corresponding primer sequences are as follows: hTERT 5′-GGCCGGGCTCCCAGTGGATTCG-3′ (−293 to −272) and 5′-CAGCGGGGAGCGCGCGGCATCG-3′ (+20 to −2); c-MYC 5′-AGGGAGAGGGTTTGAGAGGG-3′ (−401 to −382) and 5′-GAAGGTGGGGAGGAGACTCA-3′ (−272 to −291). The relative amount of PCR product amplified from the ChIP assay was normalized to the input DNA and calculated as follows: Relative amount = ([IP] – [IgG])/[Input], where [IP], [IgG], and [Input] are the relative amount of PCR products from 0.25% of input DNA ([Input]), immunoprecipitated DNA with respective antibody ([IP]), and negative control IgG ([IgG]).

### Statistics

All experiments were performed at least 3 times, and the corresponding data are shown. The results are expressed as mean ± standard error of mean. The statistical significance was determined using a two-sided Student’s *t*-test. Statistical significance was defined as P<0.05 (*P<0.05; **P<0.01; ***P<0.001).

## Results

### FOXO3a inhibits the onset of replicative senescence in HUC-F2 cells

**Figure 1:**
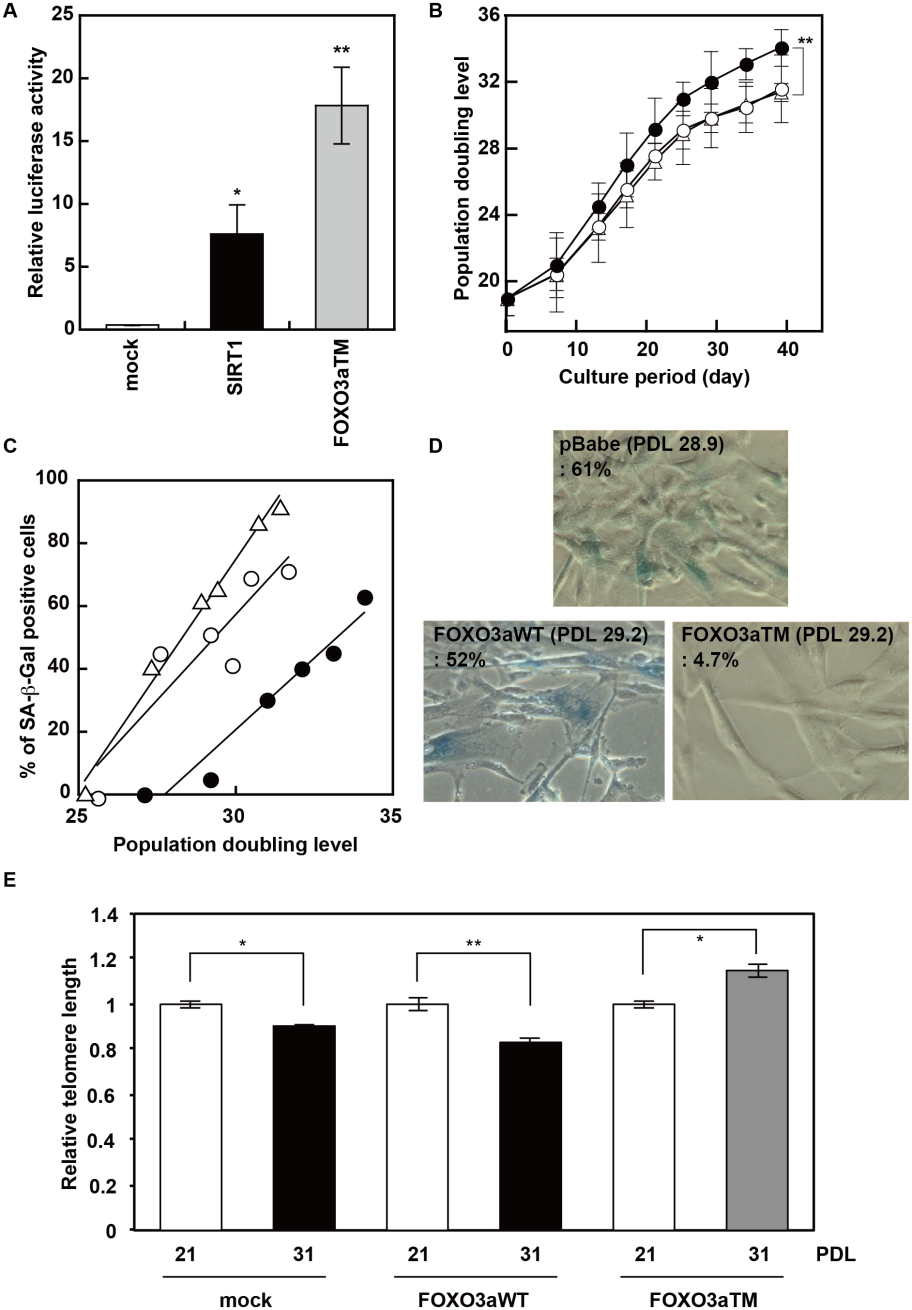
FOXO3a inhibits the onset of replicative senescence in HUC-F2 cells. A, Effect of SIRT1 on the activity of FOXO3a in HUC-F2 cells. The activity of FOXO family proteins was determined by normalized luciferase activity under the control of DBE, the consensus sequence for the FOXO family proteins. B, Effect of FOXO3a on replicative senescence of HUC-F2 cells. After HUC-F2 cells were transduced with recombinant retrovirus for FOXO3aWT (○), FOXO3aTM (•), or mock (▵), replicative potential (B) and SA-β-Gal activity (C and D) were monitored. E, Relative telomere length. Relative telomere lengths of HUC-F2 cells transduced with FOXO3aWT, FOXO3aTM or mock were determined by qPCR.

We have previously reported that SIRT1 inhibited the onset of replicative senescence in normal human fibroblast HUC-F2 cells through transcriptional activation of hTERT [6]. In this study, we aimed to identify downstream mediators of SIRT1 that function in the activation of hTERT. Here we focused on the link between SIRT1 and FOXO family proteins first described by Brunet et al [16]–[18]. They reported that SIRT1 and FOXO3 formed a complex in response to oxidative stress, and SIRT1 deacetylated FOXO3. Then, we investigated the involvement of FOXO3a in the SIRT1-induced activation of hTERT transcription. First, we used pGL3-DBE, which contains the consensus sequence (TTGTTTAC) for the forkhead family of transcription factors (FOXO), to monitor their activity [12]. Our results show that SIRT1, as well as a dominant active form of FOXO3a (FOXO3aTM), activated DBE-dependent transcription (Figure 1A), suggesting that SIRT1 activates FOXO family proteins in HUC-F2 cells.

Next, we aimed to clarify whether FOXO3a also inhibits the onset of replicative senescence in HUC-F2 cells. HUC-F2 cells were transduced with either FOXO3aWT or FOXO3aTM recombinant retrovirus or a control retrovirus and cultured for 40 days (Figure S1). Proliferative potential and senescence-associated β-galactosidase (SA-β-Gal) activity were then monitored (Figure 1B–D). Our results clearly showed that the dominant active form of FOXO3a (FOXO3aTM) significantly promoted proliferation and inhibited the onset of replicative senescence in HUC-F2 cells in a manner similar to SIRT1 [6]. Previously, we demonstrated that SIRT1 inhibits the onset of replicative senescence in HUC-F2 cells by enhancing the transcription of hTERT. Furthermore, we detected a significant elongation of telomere length in HUC-F2 cells transduced with FOXO3aTM (Figure 1E). Therefore, we next evaluated if FOXO3a, a downstream mediator of SIRT1, would exert anti-senescence function in HUC-F2 cells by enhancing hTERT transcription.

### FOXO3a enhances the transcription of hTERT

**Figure 2:**
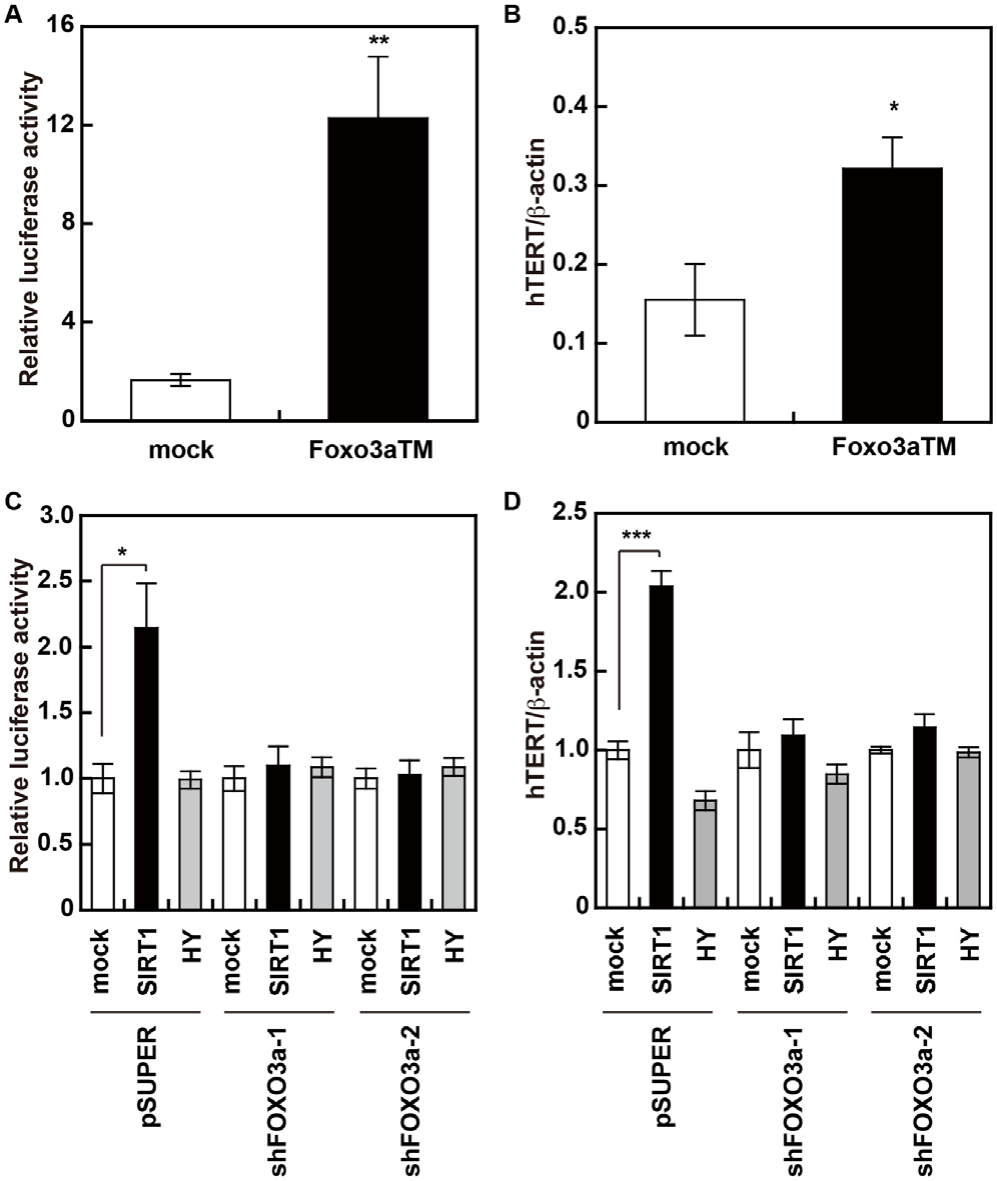
FOXO3a enhances hTERT transcription. Transcriptional activation of the hTERT gene by FOXO3a was determined by normalized luciferase activity under the control of the hTERT core promoter (A) and qRT-PCR analysis of hTERT mRNA (B). SIRT1 activates the transcription of the hTERT gene in a FOXO3a-dependent manner. FOXO3a-dependent transcriptional activation of the hTERT gene by SIRT1 was determined by normalized luciferase activity under the control of the hTERT core promoter (C) and qRT-PCR analysis of hTERT mRNA (D) in shRNA-treated HUC-F2 cells with reduced FOXO3a levels.

Results from the promoter assay and qRT-PCR show that FOXO3aTM increased hTERT transcription (Figure 2A and B). Next, in order to clarify the involvement of FOXO3a in the SIRT1-induced increase of hTERT transcription, we knocked-down FOXO3a in HUC-F2 cells by transduction with shFOXO3a-1 or shFOXO3a-2. Knockdown of FOXO3a at the mRNA level was confirmed by qRT-PCR (Figure S2). As shown in the results (Figure 2C and D), SIRT1, but not a dominant negative form of SIRT1 (SIRT1 HY), activated hTERT transcription. However, the SIRT1-induced increase in hTERT transcription was strongly suppressed in FOXO3a-knockdown HUC-F2 cells. These results suggest that FOXO3a functions downstream of SIRT1 to activate hTERT transcription.

### Molecular mechanisms of the FOXO3a-induced activation of hTERT transcription

**Figure 3:**
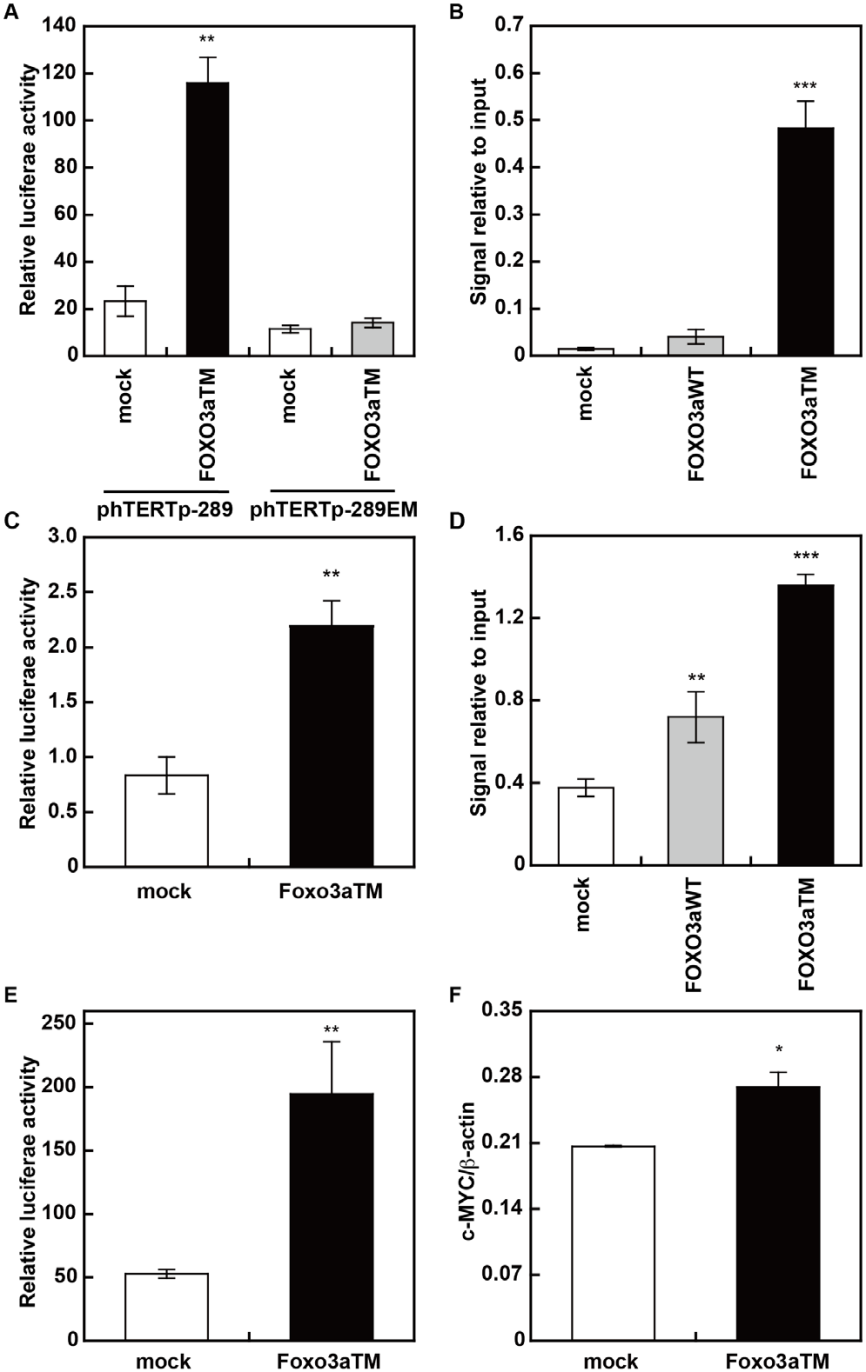
Molecular mechanism of the FOXO3a-induced activation of hTERT transcription. (A) c-MYC/E-box dependency of the FOXO3a-induced activation of hTERT transcription was assessed using luciferase reporters containing a wild-type hTERT core promoter (phTERTp-289) or a mutant hTERT core promoter (phTERTp-289EM). (B) c-MYC recruited to the hTERT core promoter was increased when HUC-F2 cells were transduced with recombinant retrovirus that expressed FOXO3aWT or FOXO3aTM. The association of c-MYC with the hTERT core promoter was assessed using a ChIIP assay. (C) FOXO3a enhances the transcriptional activation of c-MYC. GAL4-c-MYC, pFR-Luc, and the FOXO3aTM expression vector were co-transfected into HUC-F2 cells, and the normalized luciferase activity was assessed. (D) FOXO3a-induced histone H4 acetylation at the hTERT core promoter. HUC-F2 cells were transduced with recombinant retrovirus for FOXO3aWT or FOXO3aTM. The ChIP assay was performed to identify the acetylation status of histone H4 at the hTERT promoter. (E, F) The effect of FOXO3a on the transcription of c-MYC was determined by normalized luciferase activity under the control of the c-MYC promoter (E) and by qRT-PCR analysis of c-MYC mRNA (F).

Next, we investigated the molecular mechanisms of the FOXO3a-induced increase in hTERT transcription. We first examined the involvement of c-MYC, a dominant transcriptional activator for the hTERT promoter, in the enhancement of hTERT transcription. To clarify this, we employed a wild type hTERT promoter reporter vector (phTERTp-289) and a mutant hTERT promoter reporter vector with disrupted E-boxes (phTERTp-289EM) and performed a promoter assay. Our results indicate that the FOXO3a-induced increase in hTERT transcription is completely abolished with the disruption of the E-boxes (Figure 3A). This suggests that the FOXO3a-induced increase in hTERT transcription is dependent on c-MYC/E-box. In fact, we observed an increase in the amount of c-MYC recruited to the hTERT promoter by ChIP analysis using anti-c-MYC antibody (Figure 3B). Furthermore, we examined the transcriptional activation ability of c-MYC using a vector expressing c-MYC fused with the GAL4 DNA-binding domain (GAL4-c-MYC) in the promoter assay. These results demonstrate that FOXO3a significantly increased the transcriptional activation ability of c-MYC (Figure 3C). As a result, the amount of acetylated H4 histone at the hTERT promoter was significantly increased after FOXO3a transduction (Figure 3D). In addition, we examined the effects of FOXO3a on c-MYC expression and found that FOXO3a increased the promoter activity of c-MYC (Figure 3E) and the transcription of the c-MYC gene (Figure 3F). These results suggest that FOXO3a activates c-MYC expression, which, in turn, results in enhanced quantities of c-MYC at the hTERT promoter, enhanced transcriptional activation of c-MYC and, as a consequence, upregulation of hTERT gene expression.

### Identification of c-MYC promoter elements required for FOXO3a-mediated activation

**Figure 4:**
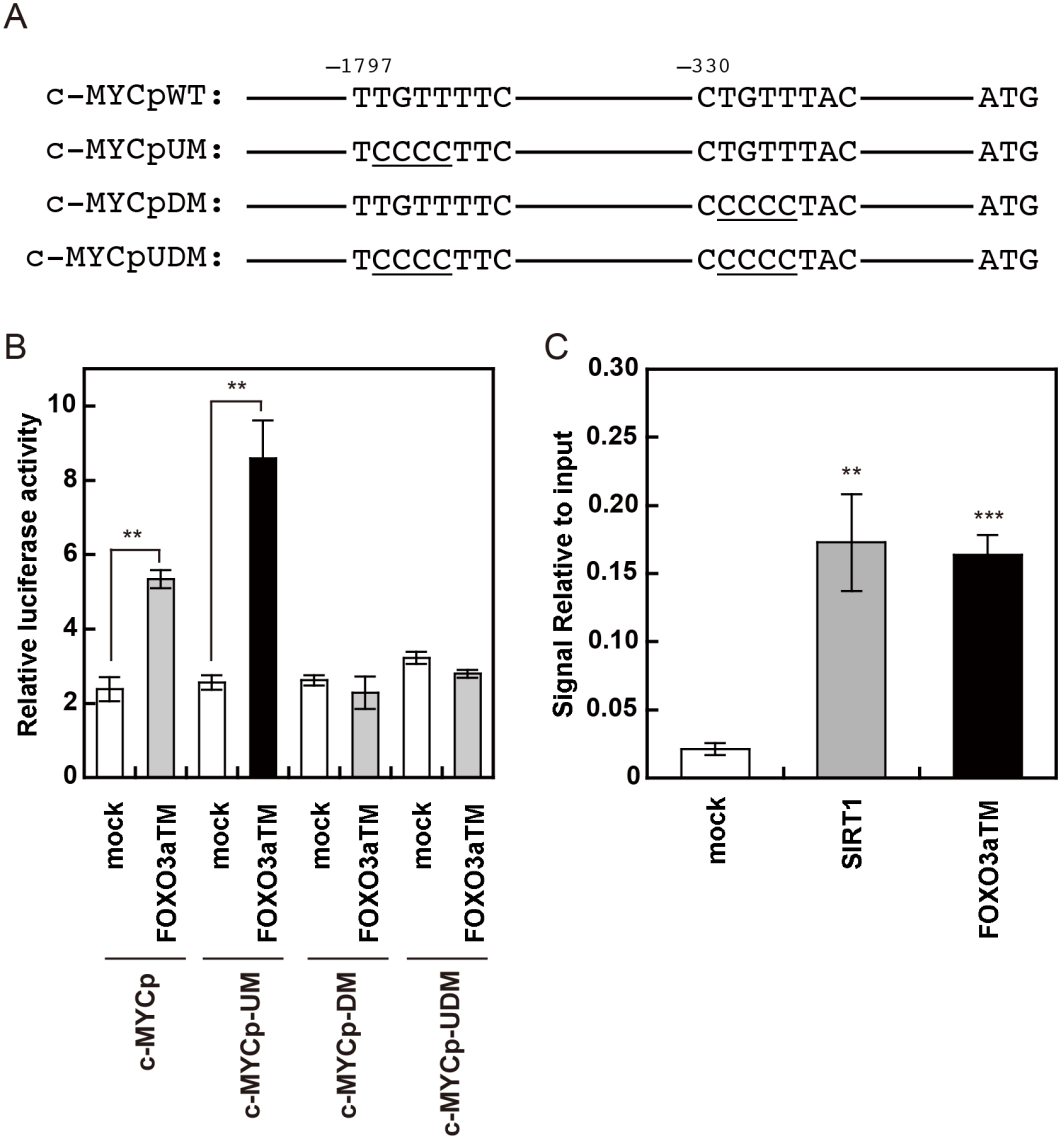
Identification of c-MYC promoter elements required for FOXO3a-mediated activation. A, Schematic representation of the human c-MYC promoter (c-MYCpWT) and its derivatives. Two putative FOXO-binding elements are shown, –1 indicates the first 5′-nucleotide from the translation initiation site, and the underlined nucleotides are those mutated for our experiments. B, Identification of elements for FOXO3a-induced activation. Elements responsible for the FOXO3a-induced activation were determined using luciferase reporters that contain a wild-type c-MYC promoter (c-MYCpWT) or a mutant c-MYC promoter (c-MYCpUM, c-MYCpDM, and c-MYCUDM). C, An increased amount of FOXO3a was recruited to the downstream region of c-MYC promoter when HUC-F2 cells were transduced with recombinant retrovirus expressing SIRT1 or FOXO3aTM. The association of FOXO3a with downstream elements of the c-MYC promoter was assessed by the ChIP assay.

**Figure 5:**
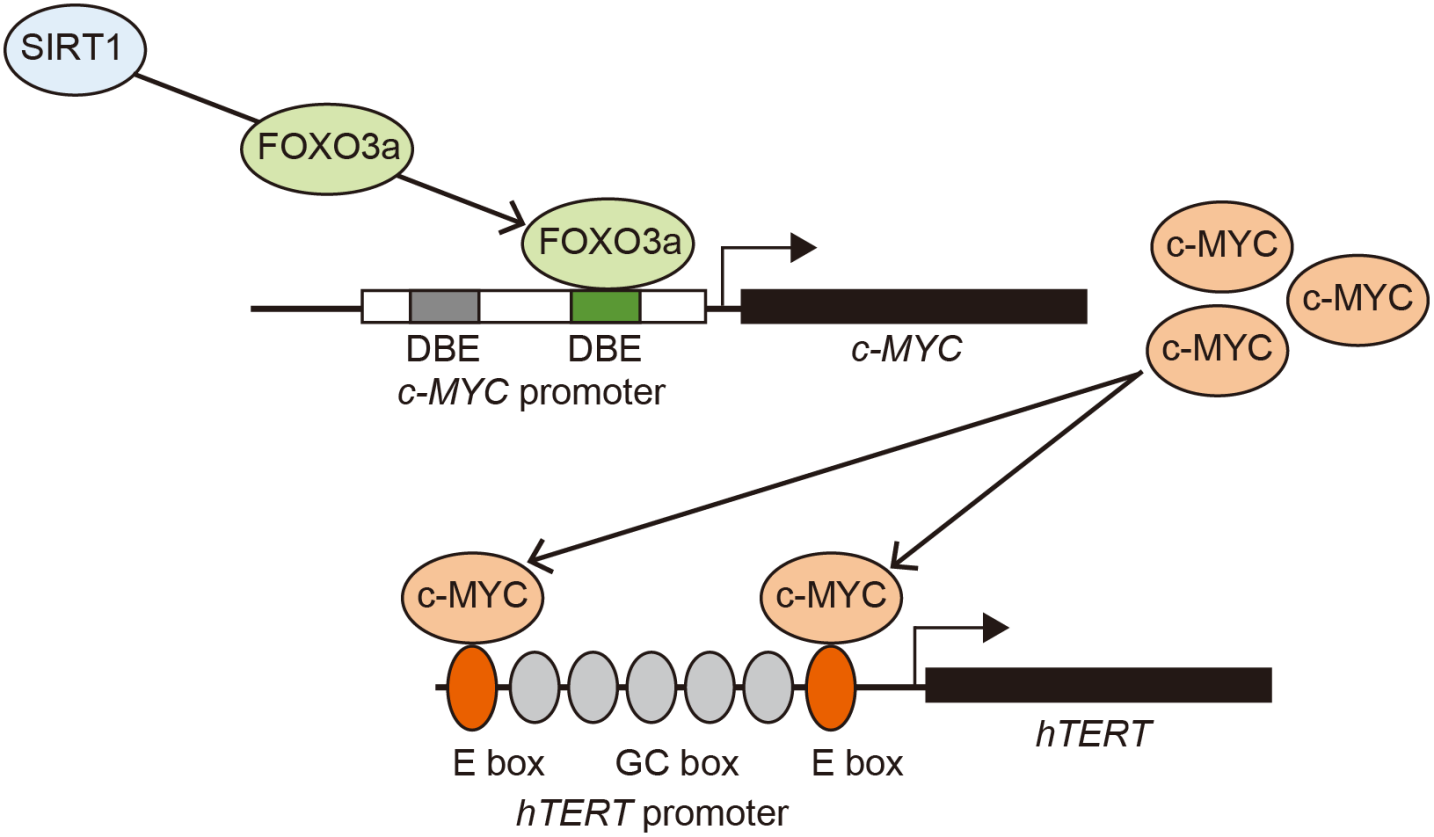
Schematic representation of FOXO3a-induced activation of hTERT transcription. FOXO3a, a downstream target molecule of SIRT1, binds to the downstream FOXO binding element (DBE) in the c-MYC promoter and activates the transcription of c-MYC, which results in the increased transcription of hTERT gene in an E-box dependent manner.

Subsequently, we investigated the FOXO3a-mediated increase in c-MYC transcription to determine if FOXO3a directly interacts and activates the c-MYC promoter. First, we searched for consensus elements for FOXO family proteins (TTGTTTAC) [12] and found two putative elements (^–1797^TTGTTTT^–1790^C and ^–330^CTGTTTA^–323^C) that resemble the consensus element in the human c-MYC promoter (Figure 4A). To assess the functionality of these elements, we generated three mutant promoters (c-MYCp UM, c-MYCp DM, and c-MYCp UDM) in which the respective elements were disrupted (Figure 4A). Our results demonstrate that the FOXO3a-induced increase in c-MYC promoter activity disappeared upon disruption of the downstream putative FOXO binding element (Figure 4B). Notably, FOXO3a, as well as SIRT1, increased the amount of FOXO3a recruited to the downstream element of the c-MYC promoter (Figure 4C, S1, and S3). Taken together, these results indicate that FOXO3a activates the c-MYC promoter through binding to the downstream FOXO binding element, which results in the increased transcription of c-MYC and consequent activation of hTERT transcription (Figure 5).

## Discussion

In our previous study, we found that SIRT1 prevented cellular senescence by enhancing the transcription of the hTERT gene [6]. Here, we investigated FOXO3a, a downstream target molecule of SIRT1 [18], [19]. Our results show that FOXO3a also prevented the induction of replicative senescence in normal human diploid cells, which is similar to reports by Kim et al. [20]. In this study, Kim et al. reported that cellular senescence was induced by the downregulation of FOXO3a expression in human dermal fibroblast. In our study, we demonstrate the novel concept that FOXO3a, as well as SIRT1, promote the transcription of the hTERT gene. Taken together, these results suggest that FOXO3a functions as an anti-senescence factor by enhancing hTERT transcription in normal human diploid cells.

In addition, we found that FOXO3a-induced enhancement of hTERT transcription is regulated in a c-MYC/E-Box dependent manner. FOXO3a augmented the amount of c-MYC recruited to the hTERT promoter and activated the hTERT promoter through c-MYC-dependent changes in histone acetylation, which likely involves c-MYC-binding to histone acetyltransferases [21], [22]. In this study, we investigated the molecular mechanisms underlying FOXO3a-induced enhancement of c-MYC transcription. To date, many different transcription factors, signal transduction pathways, and feedback loops are known to regulate c-MYC transcription [13]. Some researchers have reported that FOXO3a counteracts c-MYC through several mechanisms in which antagonists, microRNAs, and other signaling cascades participate [23]–[27]. However, in this study, by employing a mutant c-MYC promoter, we found that FOXO3a binds to a specific binding element in the c-MYC promoter and augments its transcription. The physical binding of FOXO3a to this element was confirmed by ChIP analysis. Taken together, these results indicate that anti-aging signals triggered by calorie restriction and SIRT1 are mediated by FOXO3a, which enhances c-MYC transcription by binding to a specific element in the c-MYC promoter. The different consequences might be elicited by activation of FOXO3a in a cell-type and cell-context dependent manner. In the present study, FOXO3a-dependent activation of proliferation and c-MYC transcription were observed in HUC-F2 cells, and thus might be specific events for normal human cells.

Enhanced c-MYC, in turn, augments the transcription of hTERT, which exerts molecular anti-senescence effects. Previous work has shown that the constitutive expression of TERT in transgenic mice improves epithelial barriers and produces a systemic delay in aging [28]. Furthermore, hTERT is known to possess several functions in addition to maintaining telomere length [29]. Therefore, future studies will focus on investigating hTERT-induced cellular phenotypes in an anti-aging context.

## Supporting Information

Figure S1 **FOXO3a expression level in recombinant HUC-F2 cells.**
(PDF)

Figure S2 **FOXO3a expression level in HUC-F2 cells transduced with shRNA against FOXO3a.**
(PDF)

Figure S3 **SIRT1 expression level in recombinant HUC-F2 cells.**
(PDF)
