# Supplementary material for: FOXO3a Potentiates hTERT Gene Expression by Activating c-MYC and Extends the Replicative Life-Span of Human Fibroblast
Source: PLoS One. 2014 Jul 7;9(7):e101864. doi: 10.1371/journal.pone.0101864 (PMC4085005; doi:10.1371/journal.pone.0101864)
Supplement: Figure S1 — FOXO3a expression level in recombinant HUC-F2 cells. (PDF) [file pone.0101864.s001.pdf]

Supplemental Data

Figure S1

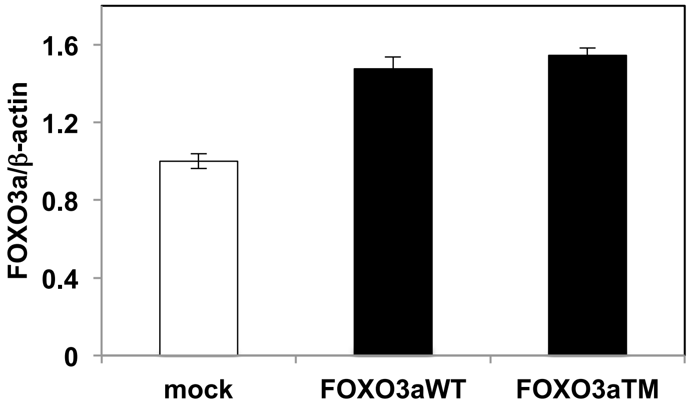

Fig. S1. FOXO3a expression level in recombinant HUC-F2 cells. The FOXO3a expression in HUC-F2 cells transduced with FOXO3aWT, FOXO3aTM or mock were assessed by qPCR in triplicate using primers specific for FOXO3a (5′-TTGAAGCGGATGCCCAAATAA-3′ and 5′-ACGTATTTCTGGAGTGTGGCAAGAG-3′) and for β-actin, and normalized to the corresponding β-actin level.
