# Supplementary material for: FOXO3a Potentiates hTERT Gene Expression by Activating c-MYC and Extends the Replicative Life-Span of Human Fibroblast
Source: PLoS One. 2014 Jul 7;9(7):e101864. doi: 10.1371/journal.pone.0101864 (PMC4085005; doi:10.1371/journal.pone.0101864)
Supplement: Figure S2 — FOXO3a expression level in HUC-F2 cells transduced with shRNA against FOXO3a. (PDF) [file pone.0101864.s002.pdf]

Supplemental Data

Fig. S2.

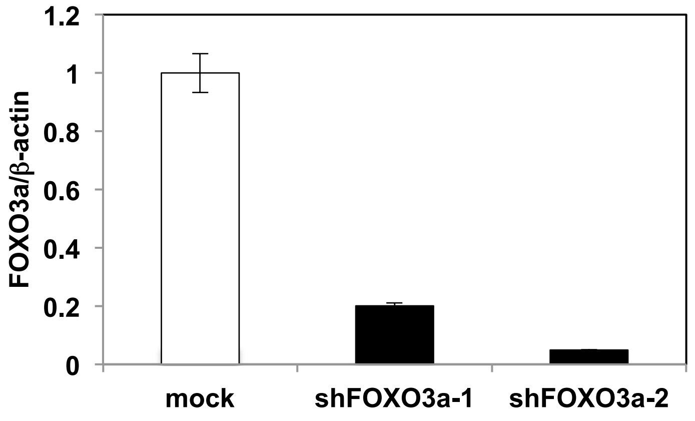

Fig. S2. FOXO3a expression level in HUC-F2 cells transduced with shRNA against FOXO3a. The FOXO3a expression in HUC-F2 cells transduced with shFOXO3a-1, shFOXO3a-2 or mock were assessed by qPCR as described above.
